# Hypertrophic lichen planus-like atopic dermatitis: a case report

**DOI:** 10.1186/1939-4551-8-S1-A214

**Published:** 2015-04-08

**Authors:** Maria Eduarda Pontes Cunha De Castro, Karine Boufleur, Phelipe Souza, Thais Nociti, Janaina M Melo, L Karla Arruda, Renata Nahas

**Affiliations:** 1 Ribeirão Preto Medical School, University of São Paulo, Brazil; 2 Clinical Hospital of Ribeirão Preto Medical School, Brazil; 3 Brazilian Association of Allergy and Immunology, Brazil

## Background

Atopic dermatitis (AD) is a chronic inflammatory skin disease characterized by pruritic eczematous lesions, which can be associated with other allergic comorbidities. Differential diagnosis of AD includes nummular eczema and lichen planus.

## Methods

Case report of a twenty five year-old woman with dried and scaly skin lesions, associated with itching and recurrent skin infections.

## Results

The lesions started at age 18 during the patient´s first pregnancy and had a predominant flexural distribution pattern. At the physical exam, the patient presented with erythema and infiltrative lesions in forearm and periorbital regions, and scaly erythematous papules, especially in inferior extremities. Some of these lesions presented an ulcerous center and had linear form. The patient had high specific serum IgE levels for house dust mites (*Dermatophagoides farinae* and *D. pteronyssinus*), grass and dog; sea food, fish, soy, wheat and latex. Serum total IgE was 11,100kU/L, and serology for hepatitis B and C was negative. The patient was treated with antihistamines, topical emollients and oral corticosteroids with low improvement. Because of this atypical presentation, other differential diagnoses were considered, including hypertrophic lichen planus. Skin biopsy was performed, showing hyperkeratosis, sub-acute spongiotic dermatitis and moderate acanthosis, compatible with atopic dermatitis and an evolution to lichen simplex chronicus.

## Conclusions

Hypertrophic lichen planus is one of the differential diagnosis of atopic dermatitis and it commonly involves the flexor surfaces of the extremities bilaterally. Adult-onset AD can present with non-typical morphology and localization, therefore it is important to distinguish these two entities, since lichen planus can be associated with other diseases, such as viral hepatitis.

## Consent

Written informed consent was obtained from the patient for publication of this abstract and any accompanying images. A copy of the written consent is available for review by the Editor of this journal.

